# Caring for the Aging: A Social Exchange Perspective on Taiwan’s Shared Care Model

**DOI:** 10.1093/geroni/igaf122.3390

**Published:** 2025-12-31

**Authors:** Shu-Chuan Jennifer Yeh, Ying-Ying Lo

**Affiliations:** National Sun Yat-sen University, Kaohsiung, Kaohsiung, Taiwan (Republic of China); I-Shou University, Kaohsiung, Kaohsiung, Taiwan (Republic of China)

## Abstract

The global population aged 60 and above is expected to grow by 34%, reaching 2.1 billion by 2050 (WHO, 2020), bringing significant challenges to families, healthcare systems, and governments. As aging populations face rising rates of chronic diseases such as cardiovascular conditions, diabetes, and dementia, the demand for long-term care increases, straining healthcare infrastructures globally. In Taiwan, approximately 840,000 individuals require long-term care, affecting 11% of families. Care responsibilities often fall on family members or outsourced caregivers, leading to financial and emotional strain, a trend observed in many countries facing similar challenges. To address this, the Taiwanese government has implemented a shared caregiving policy, allowing multiple patients to share one caregiver. This model reduces financial burdens on families and provides caregivers with shift-based work to prevent burnout, rather than continuous 24-hour care. Personal caregivers typically cost NT$2,400 per day, creating financial stress for families, while caregivers often face exhaustion. Using social exchange theory (Cook, 1987; Thibaut & Kelley, 1959), this study evaluates the shared care policy’s impact, revealing high satisfaction rates among registered nurses (88%), nursing aides (81.5%), and families (82%). The policy subsidizes caregiving costs, reduces stress, and improves job satisfaction for healthcare professionals. By balancing the costs and rewards for caregivers and families, the policy fosters a fairer exchange of resources and labor. While the policy has addressed caregiver shortages and improved caregiving standards, sustainable government funding remains essential for its long-term success. Future efforts should focus on ensuring financial stability and refining the policy.

